# Novel pruning and truncating of the mixture of vine copula clustering models

**DOI:** 10.1038/s41598-022-24274-7

**Published:** 2022-11-17

**Authors:** Fadhah Amer Alanazi

**Affiliations:** Department of Mathematics and Sciences, Prince Sultan University, 11586 Riyadh, Saudi Arabia

**Keywords:** Environmental sciences, Planetary science, Diseases

## Abstract

The mixture of the vine copula densities allows selecting the vine structure, the most appropriate type of parametric marginal distributions, and the pair-copulas individually for each cluster. Therefore, complex hidden dependence structures can be fully uncovered and captured by the mixture of vine copula models without restriction to the parametric shape of margins or dependency patterns. However, this flexibility comes with the cost of dramatic increases in the number of model parameters as the dimension increases. Pruning and truncating each cluster of the mixture model will dramatically reduce the number of model parameters. This paper, therefore, introduced the first pruning and truncating techniques for the model-based clustering algorithm using the vine copula model, providing a significant contribution to the state-of-the-art. We apply the proposed methods to a number of well-known data sets with different dimensions. The results show that the performance of the individual pruning and truncation for each model cluster is superior to an existing vine copula clustering model.

## Introduction

Model-based clustering for unsupervised learning using finite mixture models has received growing interest for decades. Finite mixture models assume that the data are generated from a mixture of *g* components. Each observation has a probability of belonging to one of these components. In the literature, finite mixture models are commonly used in many areas (see, for example^1–3^). Recently, the mixture of vine copula models received increasing interest in the literature for several reasons. First, the vine copula is a multivariate extension of the copula model using conditional densities. Therefore, copula models allow one to model the marginal distributions independently from the dependence patterns. Hence, one can fit different parametric shapes of the marginal distributions for each variable. Second, the vine copula models work on two variables at a time; hence, no restriction on the type of the bivariate copulas for each pair of variables. Thus, different types of bivariate copulas can be fitted to capture a wide range of complex dependence structures, including symmetric and asymmetric dependence shapes. Therefore, each mixture component has its flexible density. In the literature, the first attempt to incorporate the vine copula models into the finite mixture model is the work of^4^. Kim et al.^4^ introduce the mixture of (Drawable vine copula) D-vine copula densities, where the vine structure is fixed for all mixture components, and one type of the bivariate copula was fitted to all pairs of variables. Roy and Parui^5^ established a mixture of the vine copula models using a small number of the bivariate copula types and restricted their work to a sub-class of the vine copula model. Alanazi^6^ extended the work of^4^ into two-folds. First, the author extends the model from a mixture of D-vine to a mixture of regular vine (R-vine) copula model. The R-vine copula model is a general class of vine copula models that allow for a free vine structure. Second, the author fits a wide range of bivariate copula types. However, the author keeps the vine structure fixed among all the mixture components. Recently^7^, introduced a model-based clustering algorithm with a vine copula model that allows the vine structure to vary from one mixture component to another. Their method contains five main steps. In the first step, the fast clustering such as k-means of^8^ is used for the initial data clustering. In the second step, the truncated (at the first tree (level)) vine copula model is fitted and estimated for each cluster data. The *n*-dimensional vine copula model is called truncated at level ${\mathcal {T}}$ if all conditional bivariate copulas after level ${\mathcal {T}}$ are set to the independent copulas. Truncated the vine copula at the first level yields a Markov tree model. The aim of truncating the vine copula model is to reduce the computation complexity in high-dimensional cases. In the third step, the model parameters are estimated using the Expectational Conditional Maximization algorithm (ECM algorithm) of^9^, keeping the marginal distribution, bivariate copulas, and vine structures fixed based on the selection in the second step. Hence, the iteration steps of the ECM work on the Markov tree instead of the full vine copula (no-truncation level) model to reduce the model computational complexity. In the fourth step, the data is regenerated based on the successive steps of the ECM algorithm. In the final step, a full vine copula model is fitted to the final clustered data, where the marginal distribution, bivariate copulas, and vine structure of each cluster are updated. Regardless of the flexibility of their method, a mixture of Markov trees does not provide a starting value for the model’s parameters at the remaining vine trees. Therefore, important dependence may be ignored in the estimation process. Therefore, we think the full vine copula model should be fitted to the clustered data in all steps with an individual estimation of the truncation level for each cluster. Hence, the truncation level is estimated based on the cluster data instead of the fixed prior truncation level. Alanazi^10^ incorporate the truncation method of^11^, using selection criteria such as Akaike Information Criteria (AIC) of^12^) and Bayesian Information Criteria (BIC) of^13^, into the R-vine copula mixture models, where the bivariate copulas are the mixture components. However, in the mixture of R-vine densities, the R-vine densities are the mixture components (this paper). Therefore, for the mixture of R-vine densities, the truncation level should be determined individually for each cluster. In addition, AIC is known to select a complex model (see^14–16^). BIC has two drawbacks. It can select the true model if the number of the possible parameters increases sufficiently slowly with the sample size, and it assumes that all the models are equally likely^17^. Therefore, identifying the optimal truncation level for each cluster is needed. It can provide numerous flexibility to the mixture vine copula models. In addition, for the nun-truncated levels, pruning each cluster will add extra reduction to the mixture of the vine copula densities, especially in high-dimensional applications. The pruning method aims to fit independent copulas to all pairs of variables with weak/independent dependence structures. To the best of the author’s knowledge, individual pruning and truncating vine copula model of each mixture component do not exist in the literature. Therefore, this present research provides a novel method and a great contribution to state-of-the-art. For the pruning vine copula model, we apply the independent test using Kendall’s tau of^18^. For the truncation, we adopt the truncation technique of the^17^ into the mixture content. We conducted a comprehensive real-data study to illustrate the performance of the proposed method. The results show a dramatic reduction in the number of model parameters. Furthermore, the proposed method outperforms the existing vine copula clustering model.

The remainder of the paper is divided as follows. Section  introduces copula, vine copula, and model-based clustering algorithm using the vine copula model, the pruning and truncation approaches. Section  provides the result of the simulation and real-data applications. Section discusses the founding results of the studies in this paper.

## Results

In this section, we illustrate the performance of the proposed method for simulation and real data applications.

### Simulation study

**Figure 1:**
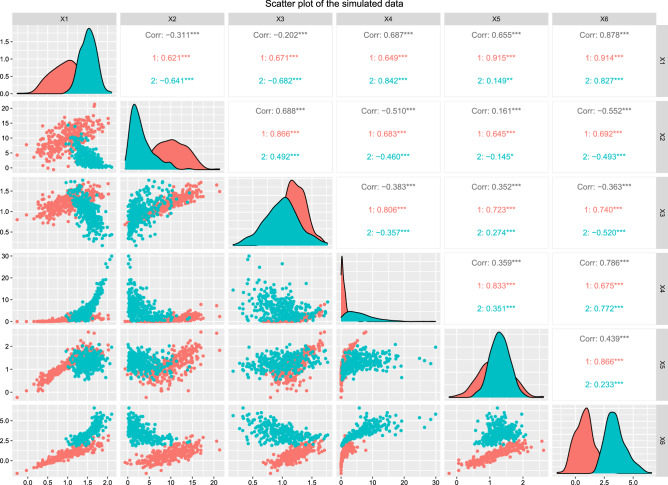
Scatter plot of the simulated data.

We simulate two mixture components from a 6-dimensional R-vine vine copula model (truncated at tree 2) with 300, and 500 observations, respectively for each cluster. The simulated data is repeated 100 times. We simulate the data using vineclust Git-hub repository of^19^. Table 1 shows the summary of the univariate marginal distributions with their corresponding parameters for each cluster. Figure 1 presents the scatter plot of the simulated data (300 observations). Listing (1) and Listing (2) present the summary of the two-component mixture of the vine copula model, where par and tau refer to copula parameter(s) and the corresponding Kendall’s tau value (the detail of the fitted models is given by RvineMatrix function of the R-program’s^20^ package VineCopula^21^).
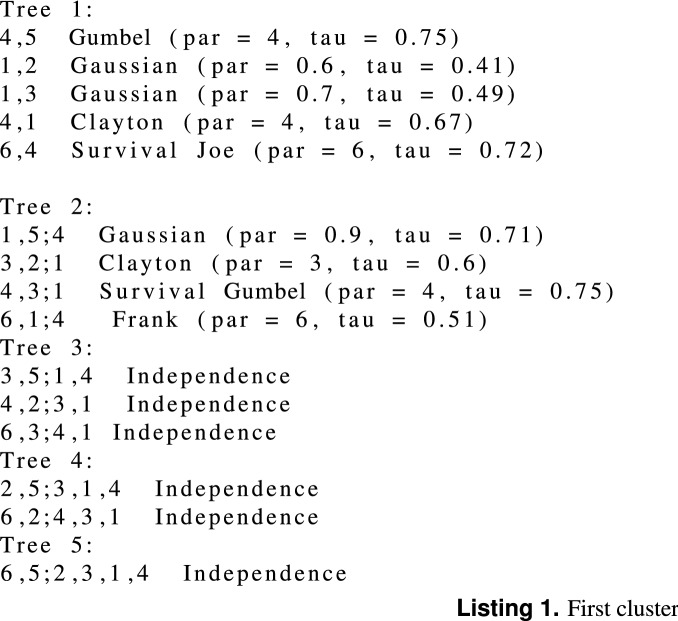

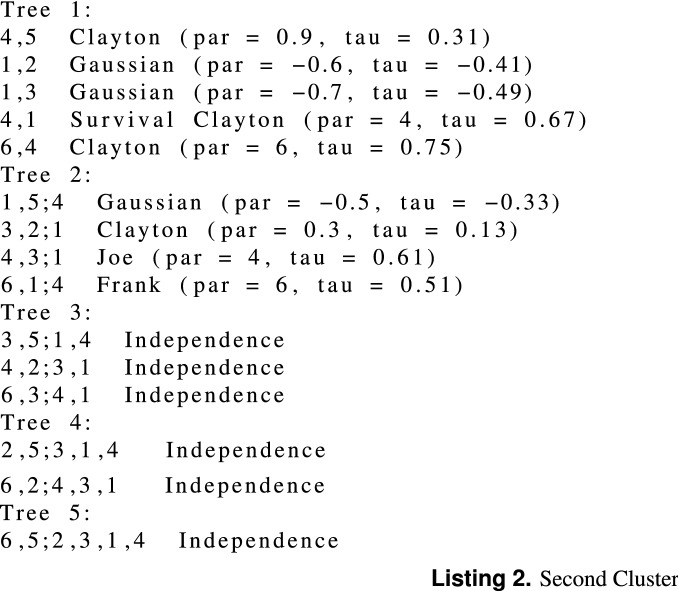

**Table 1:** Summary of the fitted univariate marginal distribution for each cluster.

Variable	First cluster $({\phi })$	Second cluster $({\phi })$
Var1	Normal (1, 0.4)	Normal (1.5, 0.2)
Var2	Normal (10, 4)	Gamma (1.5, 0.5)
Var3	Normal (1.2, 0.2)	Normal (1, 0.3)
Var4	Gamma (0.9, 0.9)	Gamma (1.5, 0.25)
Var5	Normal (1.2, 0.45)	Normal (1.3, 0.3)
Var6	Normal (0.8, 0.8)	Log-normal (1.2, 0.25)

The numbers in the bracket refer to the marginals’ parameters.

**Figure 2:**
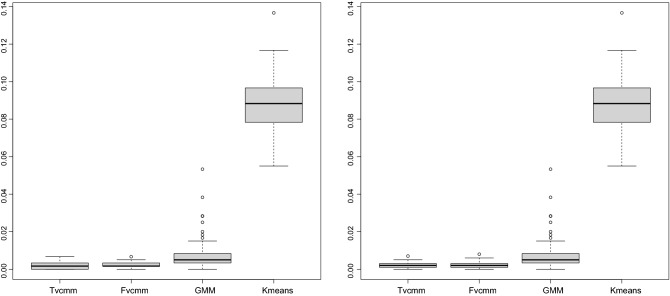
The box plot of the clustering performance of the fitted models for the simulated data (300) observations (left panel) and for the simulated data (500) observations (Right panel).

Listing (1) and Listing (2) shows the two-components $6-$dimensional vine copula mixture model. The listings show that the vine copula model for each cluster is truncated at the second tree. All the trees after the second tree are specified with independent bivariate copulas. We generated two simulated data sets from this model with 300 and 500 observations, respectively. For the sake of comparison, we fit the Gaussian finite mixture model (from^22^ package using the default setting of the package), Tvcmm, Fvcmm, and k-means. Tables 2 and 3 summarize the performance of each fitted model for the simulated data set with 300 observations and 500 observations , respectively. The best-fit model is shown in bold text. Figure 2 shows the box plots of the fitted models for each simulated data set.

**Table 2:** The summary of the performance of the Tvcmm and Fvcmm, GMM, and k-means methods fitted to the simulated data with 300 observations.

Criteria	GMM	Tvcmm	Fvcmm	k-means
Miscassification rate	0.00765	**0.00168**	0.00217	0.08821667
Average BIC	**− 5372.103**	3686.947	3589.848	–

Tvcmm, Fvcmm, GMM, and k-means refer to the truncated vine copula mixture model, full vine copula mixture model, Gaussian mixture model and the k-means method, respectively.

**Table 3:** The summary of the performance of the Tvcmm and Fvcmm, GMM, and k-means methods fitted to the simulated data with 500 observations.

**Criteria**	GMM	Tvcmm	Fvcmm	k-means
Miscassification Rate	0.00529	**0.00185**	0.00202	0.08621
Average BIC	**− 8818.664**	6013.238	5877.62	–

Tvcmm, Fvcmm, GMM, and k-means refer to the truncated vine copula mixture model, full vine copula mixture model, Gaussian mixture model and the k-means method, respectively.

### Real-data application

To test the performance of the proposed method, we applied it to several real data sets, namely diabetes, Banknotes, Flea and Sonar data sets. Table 3 summarizes the results of the truncated mixture of vine copula models and the full models. The better performance is shown in bold text.

**Table 4:** The summary of the performance of the Tvcmm and Fvcmm, GMM, and k-means methods fitted to the simulated data with 500 observations. Tvcmm, Fvcmm, GMM, and k-means refer to the truncated vine copula mixture model, full vine copula mixture model, Gaussian mixture model and the k-means method, respectively. *d*, ${\mathcal {T}}_{1}$, ${\mathcal {T}}_{2}$, ${\mathcal {T}}_{3}$, and $\delta$ refer to the data dimension (without the class variable), the truncation level of the first cluster, the truncation level of the second cluster, the truncation level of the third cluster, and the total number of the estimated model parameters, respectively.

Data (d)	Model	Misclassification rate	Average BIC	${\mathcal {T}}_{1}$(${\mathcal {T}}_{2}$) (${\mathcal {T}}_{3}$) ($\varvec{\delta }$)
Diabetes (3)	Tvcmm	0.179	4756.75	**1 (1)(1)(32)**
	Fvcmm	0.179	4773.05	NA(34)
	GMM	**0.137931**	**− 4751.316**	–
	k-means	0.179	–	–
Banknote (6)	Tvcmm	**0.005**	1674.75	**1 (1)(-)(40)**
	Fvcmm	**0.005**	1696.63	NA(63)
	GMM	**0.005**	**− 1717.445**	–
	k-means	0.04	−	–
Flea (6)	Tvcmm	0.0405	2791.96	**1(1)(1)(51)**
	Fvcmm	0.0405	2893.71	NA(90)
	GMM	**0**	**− 2785.572**	–
	k-means	0.02702	−	–
Sonar (60)	Tvcmm	**0.197**	**− 40711.15**	**1(3)(-)(522)**
	Fvcmm	0.457	− 30501.02	NA(4052)
	GMM	0.35096	33500.22	–
	k-means	0.20673	–	–

Significant values are in bold.

## Discussion

This paper incorporates the pruning and truncation methods with the vine copula model-based clustering algorithm. The pruning pairs and truncation levels are determined individually for each cluster. To illustrate the performance of the proposed method, we apply it to a simulation and real data sets. We evaluate the performance of the newly proposed method (Tvcmm), the Fcvmm algorithm of^7^, the Gaussian mixture model (GMM), and k-means. Figure 2 shows the Box plots of the misclassification rate of each algorithm per simulation replication data set. Tables 2 and 3 summarize the performance of each algorithm per simulated replication. The performance evaluation of the fitted model for the real data setes is summarized in Table 4 Lower BIC value or misclassification rate are used as a selection criterion for better clustering assignment.

For the simulated data sets, Fig. 2 shows that the Tvcmm, Fvcmm, and GMM provide a better fit than the k-means algorithm. Also, the figure shows that the performance of the Tvcmm and Fvcmm algorithms is noticeably close to each other, and both models provide a better fit than the GMM algorithm. Regarding the misclassification rate, the Tvcmm model provides better clustering assignment, while k-means is the worst. One can notice that although the misclassification rate of the Tvcmm is lower than the Fvcmm model, overall, the accuracy rate of both models is close to each other. The main reason of almost similar performance of Tvcmm and Fvcmm models, is that the data is only truncated at the second tree level. Hence, the performance of the Fvcmm algorithm, with the vine copula model truncated at the first tree level at the initial step, is close to the Tvcmm (truncated at the second tree level). Therefore, this illustrates that the truncation tree level of the data influences the final result, which is illustrated in the real data studies. From Table 4, for the small dimensional data sets, namely, Diabetes, Banknote, and Flea, the performance of Tvcmm, Fcvmm, and GMM are either identical or almost the same. However, GMM outperforms all the other algorithms for BIC value and accuracy rate for the Diabetes and Flea data sets. For Diabetes, the GMM model results in $86.21\%$ misclassification accuracy and with BIC of − 4751.316. In the case of the Flea data set, the GMM model’s performance results in a $100\%$ accuracy rate with a BIC of − 2785.572. As a result, the accuracy classification of the Tvcmm and Fvcmm algorithms are identical for Diabetes, Banknote, and Flea data sets. The result is hardly surprising, as the truncation tree level for all latter data sets is at the first tree. Therefore, the performance of Tvcmm is identical to the one of the Fvcmm model, as both treat the data at the initial steps as Markov tree structure. However, for the Sonar data set with individual truncation level for each cluster, the Tvcmm model outperforms all the fitted models with $80.3\%$ accuracy classification and BIC of − 40711.15, while the accuracy rate of the Fvcmm, GMM, and k-mean are $54.3\%$, $64.9\%$, and $79.327\%$, respectively. In addition, the Tvcmm provides a substantial model parameter reduction, resulting in 522 model parameters instead of 4052 parameters for the Fvcmm model. The result of the real data applications strongly supports this paper’s contribution and goal. From the result, the conclusion can be summarized into two main points based on the strength of the dependency among variables as follows:

- If the data exhibit weak/independent conditional dependency structure among variables after the first tree, then truncating the vine copula model at the first tree level will not affect the final result. Therefore, the misclassification rate is identical to the one of the Fvcmm model. However, due to the pruning method, Tvcmm result in less number of the estimated model parameters (this is noticed in the result of all the real data sets). In most cases, the BIC criterion selects the model with lower parameters. Comparing the result of the Fvcmm and GMM algorithm for the Sonar data set, BIC criterion selects the Fvcmm model, while its accuracy classification is lower than that of the GMM model. Therefore, our findings support the one of^7^, that a better selection criterion than BIC value is needed for the vine copula model, which can be considered as possible future work.
- If the data exhibit a strong conditional dependency structure, with a truncation tree level that can vary from one cluster to another, then the performance of the Tvcmm is superior to other fitted models. Moreover, Tvcmm results in a dramatic reduction in the number of the estimated parameters of the model.

## Methods

Copula models have been an interesting research area for decades in several areas (see, for example^23–26^), due to Sklar’s theorem^27^.

### Theorem 1

**(Sklar’s theorem)** For any an *n*-dimensional distribution function, *H*, with marginal distributions $H_{1} = H_{1}(x_{1}), \ldots , H_{n}(x_{n})$, then there exists an *n*-dimensional copula function, $C:[0,1]^{n} \rightarrow [0,1]$, such that:

1 $$\begin{aligned} H(x_{1},\ldots ,x_{n}) = C(H_{1}(x_{1}),\ldots ,H_{n}(x_{n})), \end{aligned}$$

where ${\varvec{X}}=(X_{1},\ldots ,X_{n})^{\prime }$ is an *n*-dimensional random vector. Then the joint density function can be given by:

2 $$\begin{aligned} h({\varvec{x}}) = \prod _{i=1}^{n} h_{n}({x_{n}}) \cdot c(H_{1}(x_{1}), \ldots , H_{n}(x_{n})) , \ \ {\varvec{x}} \in {\mathcal {R}}^{n} \end{aligned}$$

Where *c* is the copula density function. If all margins are continuous, then copula is unique.

Sklar’s theorem states that one can model the joint density function as a product of the marginal’s densities and the copula density. However, multivariate copulas impose the same dependence structures among variables, and only elliptical (Gaussian and t-student) copula models can be extended to multivariate cases. Vine copula incorporates the benefit of the copula models into a multivariate context. The vine copula models back to the idea of^28^ and then received more interest development in^29^. The density of *n*-dimensional copula model can be expressed, using vine copula model, as $n(n-1)/2$ bivariate copulas (pair-copulas) densities. Bedford and Cooke^30^ represent the vine copula as an unconnected graph structure known as a regular vine copula (R-vine). Due to the decomposition of the bivariate copulas, the vine copula models allow modeling two variables at a time. Each pair of variables can be modeled with a different choice of bivariate copulas; thereby, there is no restriction on the type of dependence among variables. Following the definition of the vine copula structure in^30^, the formal definition of the vine copula structure can be given as follows:

### Definition 1

The structure ${\mathcal {V}}$ is a regular vine on *n* variables if it meets the following conditions:

1. ${\mathcal {T}}_{1}$ is a tree with node set $V_{1} = \{1,\ldots ,n \}$, and edge set $E_{1} = n-1$.
2. For $i=2,\ldots ,n-1$, ${\mathcal {T}}_{i}$ is a tree with node set $V_{i} = E_{i-1}$.
3. Two edges in ${\mathcal {T}}_{i}$ become a node in ${\mathcal {T}}_{i+1}$, if and only if they shared a common node in ${\mathcal {T}}_{i}$. This condition is known as proximity condition.

The structure ${\mathcal {V}} = ({\mathcal {T}}_1, \ldots ,{\mathcal {T}}_i)$ is called a vine structure. If each edge in ${\mathcal {V}}$ is associated with a bivariate copula, then ${\mathcal {V}}$ is called a vine copula model or a pair-copula construction. The general class of the vine copula model is known as regular vine copula (R-vine copula). In the R-vine copula, there is no restriction on the way of connecting the variables. Variables can be connected by any possible shape following the three conditions given in Definition 1. There are two other sub-classes of the vine copula model, known as Canonical vine (C-vine) and Drawable vine (D-vine). These two sub-classes require a specific structure of the ${\mathcal {V}}$. For the C-vine, the variables at the first tree are connected concerning a particular variable; hence, it has a star shape. In the D-vine copula, the variables are connected sequentially, one variable after the other, taking a path shape. An example of a mixture of C-vine and D-vine copula is given in Example 2. For full details of the two sub-classes of the R-vine copula, we refer the reader to^31^. In Example 1 we introduce a simple 3-dimensional C-vine copula models (for 3-dimensional data set, the C-vine and D-vine copula models have the same vine structure).

### Example 1

(Example of 3-dimensional C-vine copula model). Suppose a 3-dimensional random vector ${\varvec{X}} = (X_{1}, X_{2}, X_{3})^{\prime }$ is given, where all the variables are continuous. Suppose further that $H_{1}, H_{2}, H_{3}$ are the corresponding univariate marginal distributions with their marginal density functions, $h_{1}, h_{2}, h_{3}$, and corresponding parameters $\varvec{\phi }_{1}, \varvec{\phi }_{2}, \varvec{\phi }_{3}$, respectively. Then, according to Sklar’s theorem^27^. The joint density function, *h*, can be given as follows:

3 $$\begin{aligned} \begin{aligned} h({\varvec{x}}; \varvec{\alpha })&= c_{3,2}(H_{3}(x_{3}; \varvec{\phi }_{3}), H_{2}(x_{2};\varvec{\phi }_{2}) ;\varvec{\theta }_{3,2}) \cdot c_{2,1}(H_{2}(x_{2}; \varvec{\phi }_{2}), H_{1}(x_{1};\varvec{\phi }_{1}) ;\varvec{\theta }_{2,1}) \\&\quad \cdot c_{3,1|2}(H_{3|2}(x_{3}|x_{2};\varvec{\phi }_{3},\varvec{\phi }_{2},\varvec{\theta }_{3,2}), H_{1|2}(x_{1}|x_{2};\varvec{\phi }_{1},\varvec{\phi }_{2},\varvec{\theta }_{1,2}) ; x_{2}, \varvec{\theta }_{3,1|2}), \end{aligned} \end{aligned}$$

where $c_{3,2}$ is the density function of the bivariate copula *c* associated with the variables 3, and 2, and $\varvec{\theta }_{3,2}$ its corresponding parameters. $c_{3,1|2}$ is the conditional density function of the conditional bivariate copula between the third and first variables conditioning on the second variable. We can see that the conditional copula, $c_{3,1|2}$ depends on the conditioning $x_{2}$. In most of the vine copula applications, and to reduce the model complexity, the $c_{3,1|2}$ assumed to be independent of the value of the $x_2$, and hence, called simplified vine copula. Then, the joint density in Eq. (3) can be rewritten as follows:

4 $$\begin{aligned} \begin{aligned} h({\varvec{x}}; \varvec{\alpha })&= c_{3,2}(H_{3}(x_{3}; \varvec{\phi }_{3}), H_{2}(x_{2};\varvec{\phi }_{2})) \cdot c_{2,1}(H_{2}(x_{2}; \varvec{\phi }_{2}), H_{1}(x_{1};\varvec{\phi }_{1})) \\&\quad \cdot c_{3,1|2}(H_{3|2}(x_{3}|x_{2};\varvec{\phi }_{3},\varvec{\phi }_{2},\varvec{\theta }_{3,2}), H_{1|2}(x_{1}|x_{2};\varvec{\phi }_{1},\varvec{\phi }_{2},\varvec{\theta }_{1,2})) \end{aligned} \end{aligned}$$

**Figure 3:**
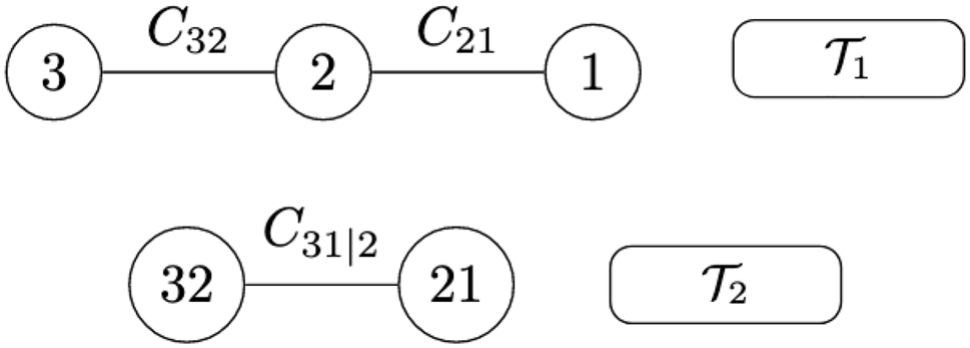
3-dimensional C-vine copula.

The structure of the 3-dimensional C-vine copula model for this example is presented in Fig. 3.

For a *n*-dimensional vine copula model, the joint density function, *h* is given as follows:

5 $$\begin{aligned} \begin{aligned} {} & h({\varvec{x}}; \varvec{\alpha }) = \prod _{j=1}^{n} h_{j}(x_{j};\phi _{j}) \\&\quad \cdot \prod _{i=1}^{n-1}\prod _{e \in E_{i}} c_{e_{m},e_{k}|D_{e}} (H_{e_{m}|D_{e}}(x_{e_{m}}|{\varvec{x}}_{D_{e}};\phi _{e_{m}|D_{e}},\varvec{\theta }_{e_{m}|D_{e}}), H_{e_{k}|D_{e}}(x_{e_{k}}|{\varvec{x}}_{D_{e}};\phi _{e_{k}|D_{e}},\varvec{\theta }_{e_{k}|D_{e}});\varvec{\theta }_{e_{m},e_{k}|D_{e}}), \end{aligned} \end{aligned}$$

where $\varvec{\alpha }$ is the parametric vector of all the model parameters, ${\textbf{x}}_{D_{e}}$ is a sub-vector of ${\varvec{x}} = (x_{1},\ldots ,x_{n})^{T} \in {\mathcal {R}}^{n}$ and $D_{e}$ is the set of the conditioning variables. At the first tree, there are no conditioning variables; hence, $D_{e}$ is an empty set in the first vine copula model. For $T_{i}, i=1,\ldots ,n-1$, $D_{e} = i-1$. $H_{e_{m}|D_{e}}$ is the conditional distribution function of $X_{e_{m}}|\varvec{X_{D_{d}}}$, with the corresponding marginal $\varvec{\phi }_{e_{m}|D_{e}}$, and the conditional bivariate copula parameters $\varvec{\theta }_{e_{m}|D_{e}}$.

### Finite mixture with vine copula model

This section will briefly introduce the model-based clustering algorithm with the vine copula model using the ECM algorithm following the work of^7^. For more details, we refer to the latter reference. In addition, we will discuss the pruning and truncation technique for the mixture of the vine copula models proposed in this paper.

### mixture of the vine copula model

Finite mixture models assume that the data are generated from a mixture of *g* components, $g=1,.., G$. Using an iterative algorithm, such as ECM, each observation is assigned to one of the mixture components with a probability. Incorporating the vine copula models into a mixture context adds numerous flexibility to the finite mixture models. The mixture of the vine copula models uncovers complex hidden bivariate dependence patterns among the variables. To define the mixture of the vine copula model formally, suppose that an *n*-dimensional random vector ${\varvec{X}} = (X_{1}, \ldots ,X_{n})^{\prime }$ is given. Suppose further that we draw *t* independent realization = $\varvec{x_{t}}=(x_{t,1}, \ldots .,x_{T,n})$, $t=1,..,T$, from ${\varvec{X}}$. Then we said that ${\varvec{X}}$ is generated from a mixture of *g*-components R-vine copula densities, if its density is given as follows:

6 $$\begin{aligned} h({{x}};\varvec{\delta }) = \sum _{g=1}^{G} \pi _{g} \cdot h_{g}({{x}};\varvec{\alpha }_{g}), \end{aligned}$$

where $\varvec{\delta }$ is the parameters vector that contains all the mixture model parameters, and $\varvec{\delta }_{g} = (\pi _{g}, \varvec{\phi }_{g}, \varvec{\theta }_{g})$ the parameters vector of all the parameters of the $g{{\rm th}}$ component. $h_{g}({{x}};\varvec{\alpha }_{g})$ is the density of the $g{{\rm th}}$ component and $\pi _{g}$ is the mixing proportion (mixture weight) that satisfies the following two conditions:

1. $$\sum _{g=1}^{G} \pi _{g} = 1$$
2. $$0< \pi _{g} < 1$$

In this paper, we will use the Inference for margins (IFM) method of^32^. The IFM is a two-stage approach. In the first step, the marginal distribution is estimated parametrically. Then, the estimated margins parameters are used to estimate the copula parameters.

The flexibility of the mixture of the vine copula models comes with the cost of the complex computational process. However, pruning and truncating the mixture vine copula models recover this limitation and provide a great parameter reduction. In this paper, we incorporate the truncation method of^17^. In^17^, the authors apply a new modified Bayesian Information Criteria (*mBICV*) of the traditional Bayesian Information Criteria (*BIC*) of^13^ to select the optimal truncation level of the vine copula model. Determining the optimal truncation level in their method start by fitting a low truncation level and calculating the mBICV. Then, gradually add more vine copula trees until there is no improvement in the mBICV value. The *BIC*, and *mBICV* can be given as follows:

7 $$\begin{aligned} BIC= & {} -2 \ln l(\varvec{{\hat{\alpha }}}) + p \ln (T) \end{aligned}$$

8 $$\begin{aligned} mBICV= & {} -2 \ln (\varvec{{{\hat{\theta }}}}) + \vartheta \ln (T) - 2 \sum _{i=1}^{n-1}(q_{i} \ln (\varphi _{0}^{i}) - (n-i-q_{i}) \ln (1-\varphi _{0}^{i}), \end{aligned}$$

where $\varvec{{\hat{\delta }}}$, is the estimated parameters of the model, *T* is the total number of observations, *p* is the total number of the model parameters, $\varvec{{{\hat{\theta }}}}$ the estimated parameters of the bivariate copulas, $\vartheta$, is the (effective) number of the model parameters, *i* is the tree level of the vine copula model, $\varphi _{0}$ is the prior probability that the bivariate copula is a non-independent copula, and $q_{i}$ is the total number of non-independent bivariate copulas in tree *i*. For the pruning method, we use the independent test based on Kendall’s tau introduced in^18^. In the following example, we will explain the idea of the mixture of the vine copula model with the pruning and truncation technique.

#### Example 2

**Figure 4:**
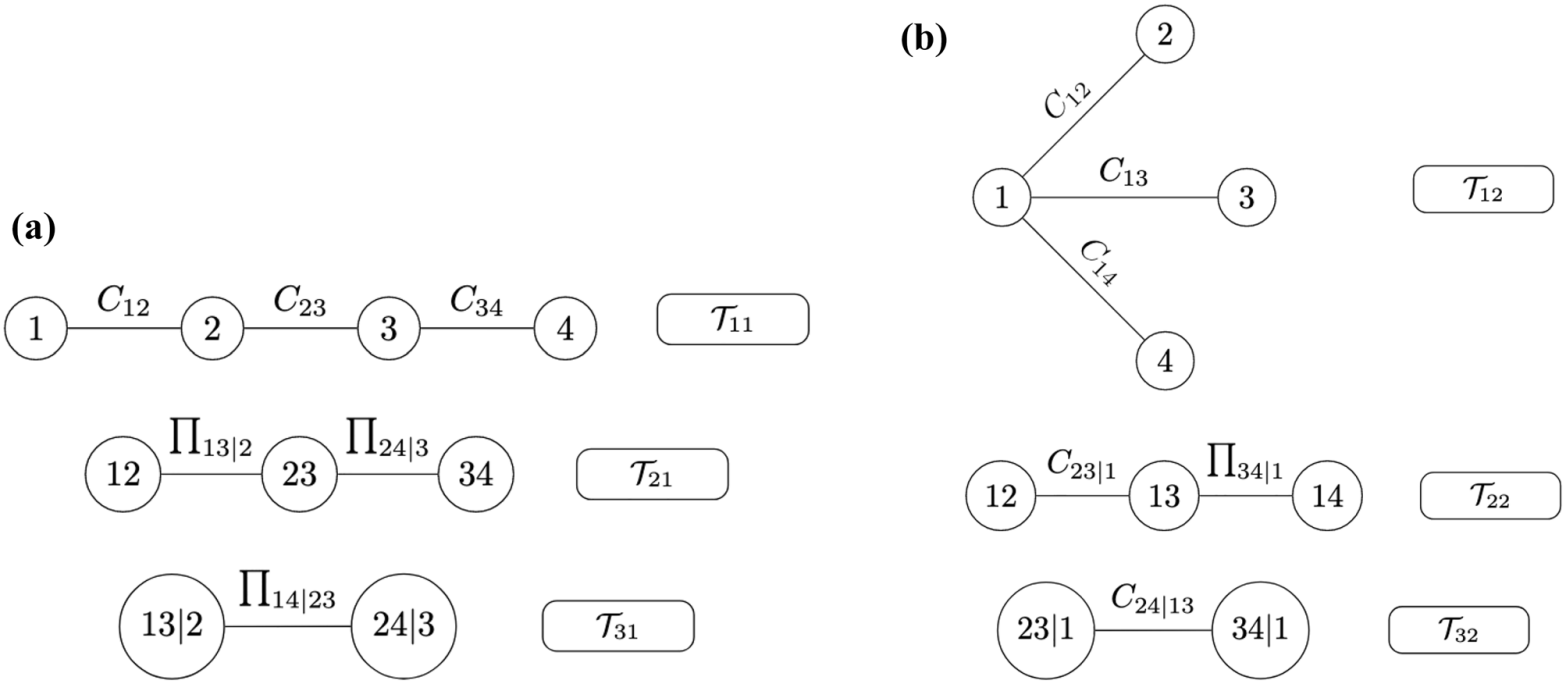
Two-component mixture of R-vine copula densities. (**a**) The left panel represents a 4-dimensional D-vine copula as the first cluster of the mixture model. (**b**) The right panel represents a 4-dimensional C-vine copula as the second cluster of the model.

(A two-components mixture of 4-dimensional vine copula mixture model). Assume that a data set is generated from two components 4-dimensional random vectors ${\varvec{X}}_{1} =(X_{11},X_{21},X_{31},X_{41})$, and ${\varvec{X}}_{2} =(X_{12},X_{22},X_{32},X_{42})$ and are given. Suppose further that *t* independent realization, $\varvec{x_{t}}=(x_{t,1}, \ldots ,x_{T,n})$, $t=1,..,T$, are drawn from $\varvec{X_1}, \varvec{X_2}$, respectively. Figure 4 represents a two-component mixture of vine copula. From the figure, one can see that each component has its own vine structure. The first component follows the D-vine copula structure, while the second component is a C-vine copula structure. ${\mathcal {T}}_{11}, {\mathcal {T}}_{12}$, and ${\mathcal {T}}_{13}$ represent the trees of the first vine structure, whereas ${\mathcal {T}}_{12}, {\mathcal {T}}_{22}$, and ${\mathcal {T}}_{32}$ refer to the trees of the second vine structure. For each vine copula model, only the first two trees are fitted with (different) bivariate copulas. For the third tree of each component, independent bivariate copulas are specified. Hence, each component is truncated at the second tree. Moreover, at the first two trees of each component, some pairs are associated with independent copulas, representing the pruning method. Then the density of the two-component mixture of the vine copula model can be given as:

9 $$\begin{aligned} h({{x}};\varvec{\delta }) = \pi _{1} \cdot h_{1}({{x}};\varvec{\alpha }_{1}) + \pi _{2} \cdot h_{2}({{x}};\varvec{\alpha }_{2}) \end{aligned}$$

$h_{1}({{x}};\varvec{\alpha }_{1}), h_{2}({{x}}; \varvec{\alpha }_{2})$ can be given in a similar way as in Eq. (5).

### ECM algorithm

The parameters of the mixture model are usually estimated using iterative methods, such as Expectation Maximization (EM) algorithm^33^ and Expected Conditional Maximum (ECM) algorithm^9^). In the general situation, estimating the model parameters can be given by computing the the value that maximizes the log-likelihood of the given data is as follows:

10 $$\begin{aligned} l(\varvec{\delta };{{x}}) = \ln \prod _{t=1}^{T} h({{x}}_{t};\varvec{\alpha }) = \ln \prod _{t=1}^{T} \sum _{g=1}^{G} \pi _{g} \cdot h_{g}({{x}}_{t};\varvec{\alpha }_{g}). \end{aligned}$$

However, since the true label of the data is unknown, the EM algorithm treats the data as incomplete data and introduces latent variables ${\varvec{z}}_{t} = (z_{t,1},\ldots ,z_{T,g})^\prime$. $z_{t,g} =1$ if the ${{x}}_{t}$ belongs to the $g^{th}$ component and $z_{t,g} =0$ otherwise, and the random vector ${\varvec{Z}}_{t}$ follows multinomial distribution, such that: ${\varvec{Z}}_{t} \sim Mult(1,(\pi _{1},\ldots ,\pi _{g}))$. Therefore, we can define the complete data as ${{x}}_{c} = ({{x}}_{t},{\varvec{z}}_{t})^{\prime }$. Hence, the log-likelihood of the complete data can be given by:

11 $$\begin{aligned} l_{c}(\varvec{\delta };{\varvec{z}},{{x}})= \ln \prod _{t=1}^{T}\prod _{g=1}^{G} \left[ \pi _{g} \cdot h_{g}({{x}}_{t};\varvec{\alpha }_{g}) \right] ^{z_{t,g}} = \sum _{t=1}^{T} \sum _{g=1}^{G} z_{t,g} \cdot \ln \pi _{g} + \sum _{t=1}^{T} \sum _{g=1}^{G} z_{t,g} \cdot \ln h_{g}({{x}}_{t};\varvec{\alpha }_{g}), \end{aligned}$$

where $h_{g}({\varvec{x}}_{t};\varvec{\alpha }_{g})$ is given in Eq. (5). EM-algorithm is commonly used in the mixture literature. The E-step computes the conditional expectation of the log-likelihood of the complete data, given the observed data at the current estimation of the model parameters. The M-step, then, maximizes the expected log-likelihood from the E-step over all the model parameters. The iterations are continuous till the model converges. However, in the vine copula model, the joint estimation of the marginal parameters, bivariate copula parameters, and mixture weight parameters of the $g^{th}$ component is not tractable and efficient^7^. Therefore, the^7^ adapted the ECM algorithm with the mixture of the vine copula models. ECM algorithm divided the M-step of the *EM* algorithm into three lower dimensional steps called CM-steps. A brief introduction, following^7^, of the CM-steps in the mixture of the vine copula models can be given as follows:

- E-step: This step calculates the posterior probability that an observation $\mathbf {x_i}$ belongs to the $g^{th}$ mixture component given the current value of the mixture weight, $\pi _{g}^{s}$, and $\varvec{\alpha }^{s}_{g}$, where *s* indicates the first iteration:
  12 $$\begin{aligned} r^{(s+1)}_{t,g} = \frac{ \pi _{g}^{(s)} \ h_{g}({\textbf{x}}_{t};\varvec{\alpha }_{g}^{(s)})}{ \sum _{g^{\prime }=1}^{G} \pi ^{(s)}_{g^{\prime }} \ h_{g}({\textbf{x}}_{t};\varvec{\alpha }_{g^{\prime }}^{(s)})} \ \end{aligned}$$
  for $$t=1,\ldots ,T$$, and $$g=1,\ldots ,G$$.
- CM-step 1: (update the mixture weights): Maximize $l_{c}(\varvec{\delta };{\textbf{z}}, {\textbf{x}})$ over the mixture weights $\pi _{g}$ given $r_{t,g}^{(s+1)}$, such that:
  13 $$\begin{aligned} \pi _{g}^{(s+1)} = arg \ max_{\pi _{g}} \sum _{t=1}^{T} r_{t,g}^{(s+1)} \ \cdot \ \ln \pi _{g} \end{aligned}$$
  A closed form solution of $\pi _{g}^{(s+1)}$ exists and can be given as:
  14 $$\begin{aligned} \pi _{g}^{(s+1)} = \frac{\sum _{t=1}^{T}r^{(s+1)}_{t,g} }{T}, \ \ \ g=1,\ldots ,G. \end{aligned}$$
- CM-step 2 (update the marginal parameters): Maximize $l_{c}(\varvec{\delta };{\textbf{z}}, {\textbf{x}})$ over the marginal parameters $\varvec{\phi }_{g}$ given the current value of the bivariate copula parameters $\varvec{\theta }_{g}^{(s)}$, and $r_{i,g}^{(s+1)}$:
  15 $$\begin{aligned} \varvec{\phi }_{g}^{*} = arg \ max _{\varvec{\phi }_{g}} \sum _{t=1}^{T} r_{t,g}^{(s+1)} \ \cdot \ \ln h_{g}({\textbf{x}}_{t}; \varvec{\phi }_{g}, \varvec{\theta }_{g}^{(s)}) \end{aligned}$$
  for $g=1,\ldots ,G$. $\varvec{\phi }_{h}^{*}$ is the optimal marginal parameter estimate of the $g^{th}$ component. Since a closed-form solution does not exist, the $l_{c}(\varvec{\delta };{\textbf{z}}, {\textbf{x}})$ is maximized numerically over $\varvec{\phi _{g}}$ such that:
  16 $$\begin{aligned} \varvec{\phi }_{g}^{(s+1)} = max _{\varvec{\phi }_{g}} \sum _{t=1}^{T} r_{t,g}^{(s+1)} \ \cdot \ \ln h_{g}({\textbf{x}}_{t}; \varvec{\phi }_{g}, \varvec{\theta }_{g}^{(s)}) \end{aligned}$$
- CM-step 3 (update the bivariate copula parameters): Similar to the marginal parameters, a closed-form solution that maximizes $l_{c}(\varvec{\delta };{\textbf{z}}, {\textbf{x}})$ 6over $\varvec{\theta }_{g}$ given $\varvec{\phi }_{g}^{(s+1)}$ and $r_{t,g}^{(s+1)}$ does not exist. Thus, $l_{c}(\varvec{\delta };{\textbf{z}}, {\textbf{x}})$ will be maximized numerically over $\varvec{\theta }_{g}$, such that:
  17 $$\begin{aligned} \varvec{\theta }_{g}^{(s+1)} = max _{\varvec{\theta }_{g}} \sum _{t=1}^{T} r_{t,g}^{(s+1)} \ \cdot \ \ln h_{g}({\textbf{x}}_{t}; \varvec{\phi }^{(s+1)}_{g}, \varvec{\theta }_{g}) \end{aligned}$$
  for $$g=1,\ldots ,G$$.

In this paper, the truncation and pruning techniques are applied to each model cluster individually. Therefore, the steps of the present work differ from the work of^7^ in the second and final step. Unlike the work of^7^, at the second step, no prior truncation level is determined. For the last step, truncation and pruning techniques are applied individually for each cluster. The steps of the proposed pruning and truncation method of this paper can be divided into the following steps:

1. Cluster the original data using k-means (other clustering methods are possible).
2. Obtains the copula data for each cluster from step 1.
3. Fit vine copula model for each cluster and determine the truncation and pruning pairs. For the vine structure and pair-copula selection, we use the Akaike Information Criteria (AIC) of^12^. AIC can be given as follows:
  18 $$\begin{aligned} AIC = -2 l (\varvec{{\hat{\theta }}}) + 2 p, \end{aligned}$$
  where $\varvec{{\hat{\theta }}}$, and *p* are the estimated parameters of the bivariate copulas and the number of the model parameters, respectively.
4. Run the ECM algorithm using the cluster data from step 1 and the vine copula model from step 2.
5. Re-clustering the data based on the ECM successive steps.
6. Fit vine copula model and determine the truncation and pruning pairs for each cluster.

To test the model performance, we use the BIC and misclassification rate. The best-fitted model is selected based on the lower BIC or misclassification rate.

## Data Availability

R software version 4.2.1 (R Development Core Team 2022) was used to implement the proposed methods. The R-package “vineclust” (https://github.com/oezgesahin/vineclust) and “rvinecopulib” were mainly used in this paper. Moreover, several dependent key packages were used, such as “mclust” and “VineCopula” packages. The Diabetes, Banknote data sets are available in the “mclust” package (https://cran.r-project.org/web/packages/mclust/vignettes/mclust.html). The flea data set is available in the “fdm2id” (https://cran.r-project.org/web/packages/fdm2id/index.html) package. The Sonar data set is available in the “mlbench” package from (https://rdrr.io/cran/mlbench/man/Sonar.html).
